# Stochastic Resonance Based Visual Perception Using Spiking Neural Networks

**DOI:** 10.3389/fncom.2020.00024

**Published:** 2020-05-15

**Authors:** Yuxuan Fu, Yanmei Kang, Guanrong Chen

**Affiliations:** ^1^Department of Applied Mathematics, School of Mathematics and Statistics, Xi'an Jiaotong University, Xi'an, China; ^2^Department of Electrical Engineering, City University of Hong Kong, Hong Kong, China

**Keywords:** stochastic resonance, spiking networks, visual perception, variance of image, contrast enhancement

## Abstract

Our aim is to propose an efficient algorithm for enhancing the contrast of dark images based on the principle of stochastic resonance in a global feedback spiking network of integrate-and-fire neurons. By linear approximation and direct simulation, we disclose the dependence of the peak signal-to-noise ratio on the spiking threshold and the feedback coupling strength. Based on this theoretical analysis, we then develop a dynamical system algorithm for enhancing dark images. In the new algorithm, an explicit formula is given on how to choose a suitable spiking threshold for the images to be enhanced, and a more effective quantifying index, the variance of image, is used to replace the commonly used measure. Numerical tests verify the efficiency of the new algorithm. The investigation provides a good example for the application of stochastic resonance, and it might be useful for explaining the biophysical mechanism behind visual perception.

## Introduction

The phenomenon of stochastic resonance, discovered by Benzi et al. (1981), is a type of cooperative effect of noise and weak signal under a certain non-linear circumstance, in which the weak signal can be amplified and detected by a suitable amount of noise (Nakamura and Tateno, 2019). Distinct biological and engineering experiments using crayfish (Douglass et al., 1993; Pei et al., 1996), crickets (Levin and Miller, 1996), rats (Collins et al., 1996), humans (Cordo et al., 1996; Simonotto et al., 1997; Borel and Ribot-Ciscar, 2016; Itzcovich et al., 2017; van der Groen et al., 2018), or optical material (Dylov and Fleischer, 2010) suggested that noise might be helpful for stimuli detection and visual perception.

As the visual perception of images of low contrast can find significance in many fields such as medical diagnosis, flight security, and cosmic exploration, theoretical research on stochastic resonance-based contrast enhancement has become an interesting but challenging topic (Yang, 1998; Ditzinger et al., 2000; Sasaki et al., 2008; Patel and Kosko, 2011; Chouhan et al., 2013; Liu et al., 2019; Zhang et al., 2019). Simonotto et al. (1997) used the noisy static threshold model to recover the picture of *Big Ben*, Patel et al. proposed a watermark decoding algorithm using discrete cosine transform and maximum-likelihood detection (Patel and Kosko, 2011), Chouhan et al. explored contrast enhancement based on dynamic stochastic resonance in the discrete wavelet transform domain (Chouhan et al., 2013), and Liu et al. (2019) applied an optimal adaptive bistable array to reduce noise from the contaminated images. It is more and more evident today that stochastic resonance can be utilized as a visual processing mechanism in nervous systems and neural engineering applications, although many theoretical and technical problems remain to be solved.

There exist at least three issues to be clarified. The first issue is about model selection. In the existing literatures, the neuron model commonly used for image enhancing is the static threshold model. Since the threshold neuron is too oversimplified to contain the evolution of the membrane voltage, a more realistic biological neuron model should be considered. The second issue is that one cannot find enough details from the existing algorithms. For example, in those algorithms, there is nearly no explanation of the choice of the critical threshold, across which the pixel value of a black–white image will switch. Note that a suitable threshold is vital for image enhancement, so the second question we have to face is what a critical threshold should be. The last issue is about the adoption of the quantifying index, which helps one to pick out an optimally detected image. A typical assumption is that one knows a clear or clean reference picture, but in most practical applications, how can one get such reference pictures especially when taking photos in darkness?

To answer the above questions, we consider an integrate-and-fire neuron network with global feedback in this paper. Our work can be divided into two parts. The first part is model preparation, where we theoretically observe stochastic resonance based on linear approximation. In the second part, by integrating all the physiological and biophysical aspects of visual perception, we propose an algorithm for boosting the contrast of an image photographed in darkness. We give a criterion for determining the critical threshold and adopt the variance of image to quantify the quality of the enhanced image. Our numerical tests demonstrate that the new algorithm is effective and robust.

## Stochastic Resonance in an Integrate-and-Fire Neuronal Network

Consider a global feedback biological network of *N* integrate-and-fire neurons (Lindner and Schimansky-Geier, 2001; Sutherland et al., 2009). The subthreshold membrane potential of each consisting neuron is governed by

(1) $$\begin{array}{l} C\frac{dV_{i}}{dt}=-g_{L}\left(V_{i}-V_{L}\right)+I_{i}\left(t\right)+Cf\left(t\right)+Cs\left(t\right),1\leq i\leq N \end{array}$$

where *V*_*i*_ is the membrane potential, *C* is the capacitance, *g*_*L*_ is the leaky conductance, *V*_*L*_ is the leaky voltage, and the external synaptic input is

(2) $$\begin{array}{l} dI_{i}\left(t\right)=C\sum_{k=1}^{p}a_{k}d\text{Ex}{\text{c}}_{n,k}\left(t\right)-C\sum_{l=1}^{q}b_{l}d\text{In}{\text{h}}_{n,l}\left(t\right) \end{array}$$

with the excitatory synaptic current *Exc*_*n, k*_(*t*) of rate λ_*E, k*_ and the inhibitory synaptic current *Inh*_*n, l*_(*t*) of rate λ_*I, l*_, both modeled as i.i.d. homogenous Poisson processes, with *a*_*k*_(1 ≤ *k* ≤ *p*) and *b*_*l*_(1 ≤ *l* ≤ *q*) denoting the efficacies for excitatory and inhibitory synapses, respectively. Assume that each neuron receives a subthreshold cosine signal, *s*(*t*) = εcos(Ω*t*), from the external environment. By “subthreshold,” it means that, in the absence of the synaptic current input (2), the membrane potential cannot cross the given spiking threshold from below (Kang et al., 2005). Here we use *V*_*r*_ to denote the resetting potential; that is, whenever the *i*th membrane potential reaches the threshold *V*_*th*_ from below, the *i*th neuron will emit a spike and then the membrane potential will be reset to *V*_*r*_ immediately. Let *t*_*i, k*_ be the *k*th spiking instant recorded from the *i*th neuron; then, the output spike train of the *i*th neuron can be described as *y*_*i*_(*t*) = ∑_*k*_δ(*t* − *t*_*i, k*_). In this network, the output spike trains from every consisting neuron are fed back to the *i*th neuron for 1 ≤ *i* ≤ *N* through the synaptic interaction.

(3) $$\begin{array}{l} f\left(t\right)=\frac{G}{N}\int_{\tau_{D}}^{\infty }d\tau \frac{\tau -\tau_{D}}{\tau_{S}^{2}}\text{exp}\left(-\frac{\tau -\tau_{D}}{\tau_{S}}\right)\sum_{n=1}^{N}y_{n}\left(t-\tau \right) \end{array}$$

Here the global feedback interaction is implemented by a convolution of the sum of all the spike trains with a delayed alpha function. We fix the transmission time delay τ_*D*_ = 1 and the synaptic time constant τ_*S*_ = 0.5. In Equation (3), the feedback strength *G* < 0 indicates inhibitory feedback, *G* > 0 represents excitatory feedback, and Equation (1) turns into a neuron array model for enhancing information transition (Yu et al., 2012) when *G* = 0.

For simplicity, let us drop the subscripts *k* and *l* in the rates and the synaptic efficacies, so λ_*E*_ = λ_*I*_ = λ, *p* = *q* and *b* = *ra*, with *r* being the ratio between inhibitory and excitatory inputs. Invoking diffusion approximation transforms the synaptic current to

$$\begin{array}{l} dI_{i}\left(t\right)=C\left(ap\left(1-r\right)\lambda dt+a\sqrt{p\lambda \left(1+r^{2}\right)}dB_{i}\left(t\right)\right) \end{array}$$

where (*B*_1_(*t*), *B*_2_(*t*), …, *B*_*N*_(*t*)) is *n* dimensional standard Brownian motions. With Equation (3) available, Equation (1) can be rewritten as

(4) $$\begin{array}{l} \frac{d}{dt}V_{i}=-\frac{1}{\tau }\left(V_{i}-V_{L}\right)+ap\left(1-r\right)\lambda \\ \;+a\sqrt{p\lambda \left(1+r^{2}\right)}\xi_{i}\left(t\right)+f\left(t\right)+s\left(t\right) \end{array}$$

where $\tau^{-1}=g_{L}/C$ and ξ_*i*_(*t*) is Gaussian white noise satisfying 〈ξ_*i*_(*t*)〉 = 0 and 〈ξ_*i*_(*t* + *s*)ξ_*j*_(*t*)〉 = δ(*s*) for 1 ≤ *i, j* ≤ *N*.

It has been shown that the firing rate is approximately a linear function of the external input near the equilibrium point (Gu et al., 2019), so we apply the linear approximation theory (Lindner and Schimansky-Geier, 2001; Pernice et al., 2011; Trousdale et al., 2012) to calculate the response of each neuron. Let μ = *ap*(1 − *r*)λ + *V*_*L*_/τ and $D=\frac{1}{2}a^{2}p^{2}\lambda \left(1+r^{2}\right)$. Regarding each neuron as linear filter of an external perturbation, we rewrite Equation (4) into Equation (5)

(5) $$\begin{array}{l} \frac{dV_{i}\left(t\right)}{dt}=-\frac{1}{\tau }V_{i}\left(t\right)+\left(\mu +{\langle f\left(t\right)\rangle }_{0}\right)+\sqrt{2D}\xi_{i}\left(t\right)\\ \;+\underset{\text{external}\;\text{perturbation}}{\underset{︸}{\left(f\left(t\right)-{\langle f\left(t\right)\rangle }_{0}\right)+s\left(t\right)}},1\leq i\leq N. \end{array}$$

**Figure 1:**
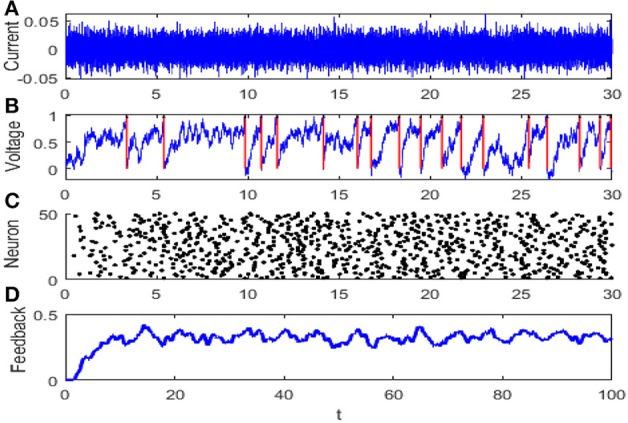
The evolution diagram of the integrate-and-fire neuron network: **(A)** diffusion approximation transforming the synaptic current with *r* = 1, **(B)** membrane potential of neuron where the red arrow denotes the discharge time, **(C)** raster plot of the network where every node denotes a spike at a corresponding time and neuron, and **(D)** feedback of the network. The parameters are set as μ = 0.8, *V*_*T*_ = 1, *V*_*R*_ = 0, *G* = 0.5, ε = 0.1, Ω = 1, τ_*S*_ = 0.5, τ_*D*_ = 1, τ_*ref*_ = 0, and *N* = 50.

For simplicity, all of the variables are dimensionless and most of parameters are taken from Lindner et al. (2005), and particularly, time is measured in unit of membrane time constant τ. The dynamical evolution of the network is illustrated in Figure 1.

**Figure 2:**
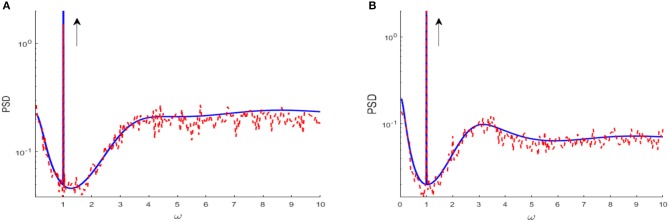
Power spectrum density obtained from linear approximation (blue solid curve), compared to simulation (red dash curve) for *D* = 0.01 **(A)** and *D* = 0.1 **(B)**, respectively. The parameters are set as μ = 0.8, *V*_*T*_ = 1, *V*_*R*_ = 0, *G* = 0.5, *N* = 3, ε = 0.1, Ω = 1, τ_*S*_ = 0.5, τ_*D*_ = 1, τ_*ref*_ = 0, and τ = 1. The black arrow indicates the spike spectral line at the driving frequency, and the remaining part characterizes the spectral density of environmental fluctuations. Clearly, the spectral line of the driving signal is riding over the fluctuation spectral density. The explanation of figure and derivation of power spectrum density are displayed in Appendix.

The phenomenon of stochastic resonance is frequently measured by the spectral amplification factor (Liu and Kang, 2018) and the output signal-to-noise ratio (Kang et al., 2005). With the help of the linear approximation theory, both the spectral amplification factor and the output signal-to-noise ratio for the homogeneous network can be explicitly attained. The spectral amplification factor is defined as the ratio of the power denoted by the delta-like spike in the output spectrum at ±Ω over the power of the input signal, namely,

(6) $$\begin{array}{l} SAF=\frac{\pi \varepsilon^{2}{\left|A\left(\Omega ,\bar{\mu },D\right)\right|}^{2}}{\left|1-GA\left(\Omega ,\bar{\mu },D\right)F\left(\Omega \right)\right|}/\pi \varepsilon^{2}=\frac{{\left|A\left(\Omega ,\bar{\mu },D\right)\right|}^{2}}{\left|1-GA\left(\Omega ,\bar{\mu },D\right)F\left(\Omega \right)\right|} \end{array}$$

while the signal-to-noise ratio, defined as the ratio of the power of the signal component over the background noise, is given by

(7) $$\begin{array}{l} SNR=\underset{\Delta \omega \to 0}{\lim}\frac{\int_{\Omega \text{-}\Delta \omega }^{\Omega +\Delta \omega }{\text{G}}_{yy}\left(\omega \right)d\omega }{S_{\text{2}}\left(\Omega \right)}=\frac{N\pi \varepsilon^{2}|A\left(\Omega ,\bar{\mu },D\right){|}^{2}}{S_{0}\left(\Omega ,\bar{\mu },D\right)} \end{array}$$

where *A* is the linear susceptibility, $\bar{\mu }$ is the base current, $F\left(\omega \right)=e^{i\omega \tau_{D}}/{\left(1-i\omega \tau_{S}\right)}^{2}$ is the Fourier transform of the kernel in Equation (2) and *S*_0_(ω, μ, *D, V*_*T*_) is the fluctuating spectral density of the unperturbed system. *G*_*yy*_(ω) is power spectral density of output spike train, which consists of the signal component *S*_1_(ω) and the fluctuation component *S*_2_(ω). Actually, within the range of linear response, the power spectrum *G*_*yy*_(ω) is a sharp power peak at the signal frequency riding over the spectral density of fluctuations, as shown in Figure 2. The detailed derivations of power spectral density *G*_*yy*_(ω), spectral amplification factor *SAF* and output signal-to-noise ratio *SNR* are further described in Appendix.

**Figure 3:**
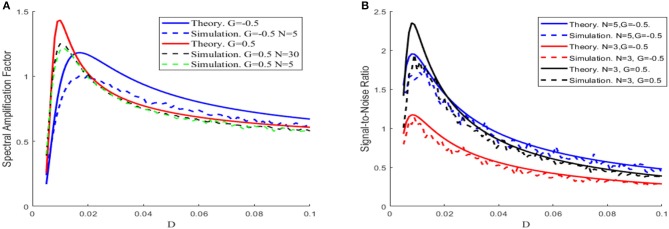
The theoretical (solid) and simulation (dash) results of spectral amplification factor **(A)** and signal-to-noise ratio **(B)** vs. noise intensity, with μ = 0.8,*V*_*T*_ = 1,*V*_*R*_ = 0, ε = 0.1,Ω = 1,τ_*S*_ = 0.5,τ_*D*_ = 1,τ_*ref*_ = 0, and τ = 1, under different sizes of the network *N* and different feedback strengths *G*, respectively.

**Figure 4:**
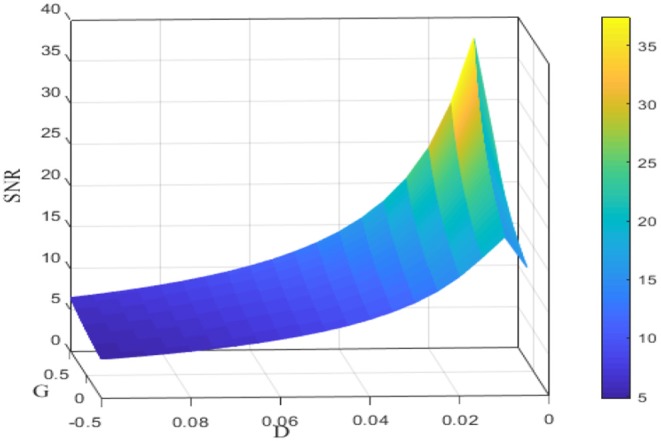
Signal-to-noise ratio for different noise intensities *D* and feedback strengths *G*. Data were obtained by the numerical simulation of a network, with μ = 0.8, *V*_*T*_ = 1, *V*_*R*_ = 0, *N* = 50, ε = 0.1, Ω = 1, τ_*S*_ = 0.5, τ_*D*_ = 1, τ_*ref*_ = 0, and τ = 1. It is clear that for fixed *G*, the signal-to-noise ratio shows a rise before fall as a function of noise intensity *D*; for fixed *D*, the signal-to-noise ratio is an increasing function of the feedback strength *G*.

**Figure 5:**
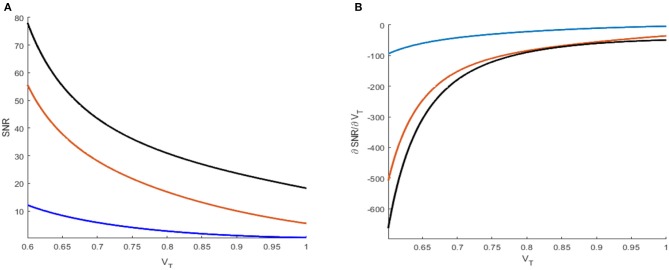
Signal-to-noise ratio **(A)** and its partial derivative with respect to threshold *V*_*T*_ **(B)** under different reference currents μ = 0.2 (blue), 0.5 (orange), and 0.8 (black). The network size is *N* = 50.

Equation (6) demonstrates that the spectral amplification factor is independent of the network size, whereas Equation (7) shows that the signal-to-noise ratio is proportional to the size. When comparing with the simulation results, Figure 3 shows that the theoretical results tend to be an overestimated approximation, but the overestimation is reduced as the network size increases. For this reason, the network size is fixed to be large enough in Figures 4, 5 so that the theoretical and simulation results are accurately matched.

Since the dependence of the spectral amplification factor or the signal-to-noise ratio on noise intensity is non-monotonic, one can conclude that stochastic resonance occurs for the given parameters in Figure 3. Figure 4 further shows the image of the signal-to-noise ratio on the two-parameter plane of noise intensity and global feedback strength. From this figure, it can be seen that, for fixed feedback strength, the existence of a sharp peak indicates stochastic resonance in the global feedback network, while for fixed noise intensity, the signal-to-noise ratio is a growing function of the feedback strength, which suggests the larger feedback strength is beneficial for resonant effect. Here we emphasize that the effect of the inhibitory feedback on the weak signal amplification is different from its effect on the intrinsic oscillation measure in Lindner et al. (2005) since these are two kinds of different synchronization. Phenomenologically, the former is the synchronization behavior of the external weak signal and the firing activity caused by noise, while the latter is the synchrony among the population neurons, and the difference in quantifying indexes directly leads to distinct observation. Thus, from the viewpoint of weak signal detection, one can say that the excitatory neural feedback is better than the inhibitory neural feedback.

Note that, in real neural activities, the spiking threshold may vary following the changing circumstance (Destexhe, 1998; Taillefumier and Magnasco, 2013), so it makes sense to consider the effect of the threshold on the population activity. By Equation (7), one has

(8) $$\begin{array}{l} \frac{\partial SNR}{\partial V_{T}}=S_{0}^{-2}N\pi \varepsilon^{2}\left(2\text{Re}\left(A^{*}\frac{\partial A}{\partial V_{T}}\right)S_{0}-|A{|}^{2}\frac{\partial S_{0}}{\partial V_{T}}\right), \end{array}$$

where

$$\begin{array}{l} \frac{\partial A}{\partial V_{T}}=\frac{i\omega }{\sqrt{D}\left(i\omega -1\right)}\left(\frac{\partial r}{\partial V_{T}}\frac{{\tilde{D}}_{i\omega -1}\left(\frac{\mu -V_{T}}{\sqrt{D}}\right)-e^{\gamma }{\tilde{D}}_{i\omega -1}\left(\frac{\mu -V_{R}}{\sqrt{D}}\right)}{{\tilde{D}}_{i\omega }\left(\frac{\mu -V_{T}}{\sqrt{D}}\right)-e^{\gamma }e^{i\omega \tau_{R}}{\tilde{D}}_{i\omega }\left(\frac{\mu -V_{R}}{\sqrt{D}}\right)}+r\frac{-\frac{1}{\sqrt{D}}\frac{\partial {\tilde{D}}_{\text{i}\omega -1}}{\partial V_{T}}{|}_{\frac{\mu -v_{T}}{\sqrt{D}}}-e^{\gamma }\frac{\left(\mu -V_{T}\right)}{2D}{\tilde{D}}_{i\omega -1}\left(\frac{\mu -V_{R}}{\sqrt{D}}\right)}{{\tilde{D}}_{i\omega }\left(\frac{\mu -V_{T}}{\sqrt{D}}\right)-e^{\gamma }e^{i\omega \tau_{R}}{\tilde{D}}_{i\omega }\left(\frac{\mu -V_{R}}{\sqrt{D}}\right)}\right)\\ \;+\frac{i\omega }{\sqrt{D}\left(i\omega -1\right)}\left(r\frac{\left(-\frac{1}{\sqrt{D}}\frac{\partial {\tilde{D}}_{\text{i}\omega }}{\partial V_{T}}{|}_{\frac{\mu -v_{T}}{\sqrt{D}}}-e^{\gamma }e^{i\omega \tau_{R}}\frac{\left(\mu -V_{T}\right)}{2D}{\tilde{D}}_{i\omega \text{-1}}\left(\frac{\mu -V_{R}}{\sqrt{D}}\right)\right)\left({\tilde{D}}_{i\omega -1}\left(\frac{\mu -V_{T}}{\sqrt{D}}\right)-e^{\gamma }{\tilde{D}}_{i\omega -1}\left(\frac{\mu -V_{R}}{\sqrt{D}}\right)\right)}{{\left({\tilde{D}}_{i\omega }\left(\frac{\mu -V_{T}}{\sqrt{D}}\right)-e^{\gamma }e^{i\omega \tau_{R}}{\tilde{D}}_{i\omega }\left(\frac{\mu -V_{R}}{\sqrt{D}}\right)\right)}^{2}}\right) \end{array}$$

and

∂S0∂VT=∂r∂VT|D~iω(μ-vTD)|2-e2γ|D~iω(μ-vRD)|2|D~iω(μ-vTD)-eγeiωτRD~iω(μ-vRD)|2        +r2Re(-1D(D~iω(μ-vTD))*∂D~iω∂VT|μ-vTD)-e2γμ-VTD|D~iω(μ-vRD)|2|D~iω(μ-vTD)-eγeiωτRD~iω(μ-vRD)|2        +r2Re((D~iω(μ-vTD)-eγeiωτRD~iω(μ-vRD))*(D~iω(μ-vTD)·∂D~iω∂VT|μ-vTD·(-1D)-eγeiωτRD~iω(μ-vRD)μ-VT2D))|D~iω(μ-vTD)-eγeiωτRD~iω(μ-vRD)|4

with Re(·) being the real part of a complex value. Here, the Whittaker notation ${\tilde{D}}_{a}$(Abramovitz and Stegun, 1964) is used for the parabolic cylinder function, with the recursion property ${\tilde{D^{\prime }}}_{a}\left(x\right)+\frac{1}{2}x{\tilde{D}}_{a}\left(x\right)-a{\tilde{D}}_{a-1}\left(x\right)=0$ and

$$\begin{array}{l} \frac{\partial r}{\partial V_{T}}=\frac{-\frac{r^{2}\sqrt{\pi }}{\sqrt{2D}}\cdot \exp\left({\left(\frac{\mu +Gr-V_{T}}{\sqrt{2D}}\right)}^{2}\right)erfc\left(\frac{\mu +Gr-V_{T}}{\sqrt{2D}}\right)}{1+\frac{Gr^{2}\sqrt{\pi }}{\sqrt{2D}}\left(\exp\left({\left(\frac{\mu +Gr-V_{R}}{\sqrt{2D}}\right)}^{2}\right)erfc\left(\frac{\mu +Gr-V_{R}}{\sqrt{2D}}\right)-\exp\left({\left(\frac{\mu +Gr-V_{T}}{\sqrt{2D}}\right)}^{2}\right)erfc\left(\frac{\mu +Gr-V_{T}}{\sqrt{2D}}\right)\right)} \end{array}$$

The evolution of the signal-to-noise ratio [Equation (7)] and its partial derivative [Equation (8)] obtained *via* the threshold is shown in Figures 5A,B, respectively. The monotonical decrease in the signal-to-noise ratio suggests that a smaller threshold is better for weak signal detection. Moreover, from these figures, one can also see that an increasing distance between the base current and the firing threshold will lead to a reduced signal-to-noise ratio, as disclosed by Kang et al. (2005). As a result, the minimum distance between the base current and the firing threshold should be an important reference in designing visual perception applications of the global feedback network.

## Stochastic Resonance Based Image Perception

We have systematically disclosed the phenomenon of stochastic resonance from the viewpoint of model investigation, and in this section, we wish to propose an algorithm for visual perception under the guidance of the above theoretical results. In fact, it is the theoretical evidence of SR in the integrate-and-fire neuron network in section stochastic resonance in an integrate-and-fire neuronal network that motivates us to do the application exploration. If noise at a certain level can amplify a weak harmonic signal *via* stochastic resonance, then noise of suitable amount can very likely enhance a more realistic weak signal such as the image of low contrast *via* aperiodic stochastic resonance.

In stochastic resonance, since the external weak signal is harmonic, one can use the spectral amplification factor or the output signal-to-noise ratio as quantifying index through frequency matching, while in aperiodic stochastic resonance, the external weak signal is aperiodic, so one has to resort to some coherence measure to describe the involved shape matching, as confirmed in neural information coding (Parmananda et al., 2005), hearing enhancement (Zeng et al., 2000). For the picture of low contrast, its contrast can be changed by noise and will attain to a maximum when the phenomenon of aperiodic stochastic resonance occurs; thus, we use the variance of image as a quantifying index as explained below. Even though a difference exists in quantifying index between stochastic resonance and aperiodic stochastic resonance, we can still use the results obtained from the model investigation as guidance. The numerical results in section stochastic resonance in an integrate-and-fire neuronal network show that positive feedback strength and low threshold are beneficial factors for observing the effect of stochastic resonance, and therefore we will take the two factors into account in the following algorithm design.

**Figure 6:**
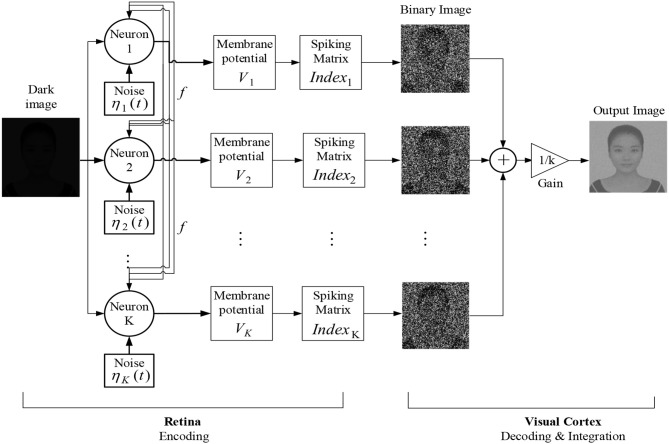
Schematic diagram of the dark image enhancement algorithm based on the global feedback integrate-and-fire network.

With the theoretical guidance in mind, we now start to present the algorithm for enhancing the image of low contrast. By the term dark image or image of low contrast, we mean that the picture is taken in a dark surrounding and cannot be detected at first sight. We put the new algorithm under the frame of the fundamental process for visual formation (Purves, 2011; Li, 2019): the photoreceptors in the retina receive the light and convert it into electrical signals, which is called encoding process, and then the signals are processed ultimately in the visual cortex, which is called decoding and integration process. Our algorithm is expounded into three steps, as shown in the flow chart in Figure 6.

### Step 1. Encoding

When light enters the eye, the retina will convert the optical signal into electrical signal first. There are two kinds of photoreceptors in the retina, which are called rods and cones, respectively. The cones are active at bright light conditions and capable of color vision, while the rods are responsible for scotopic vision but cannot perceive color. As a result, human can capture the shape of the object in dim surroundings. We use the global feedback network [Equation (5)] of *K* integrate-and-fire neurons to simulate the perceptive process for rod cells. The membrane potential $V_{i}^{m,n}$ for each neuron is governed by

(9) $$\begin{array}{l} \frac{dV_{\text{i}}^{m,n}\left(t\right)}{dt}=-\frac{1}{\tau }V_{i}^{m,n}\left(t\right)+U\left(m,n\right)+\sqrt{2D}\eta_{i}^{m,n}\left(t\right)\\ \;+f^{m,n}\left(t\right),\text{1}\leq i\leq K \end{array}$$

where the superscript corresponds to the pixels of the image and the subscript corresponds to the neurons, *U*(*m, n*)∈[0, 1] denotes the brightness of the input image, the Gaussian white noise $\eta_{i}^{m,n}\left(t\right)$ satisfying $\langle \eta_{i}^{m,n}\left(t+s\right)\eta_{j}^{m,n}\left(t\right)\rangle =\delta \left(s\right)\delta \left(i-j\right)$ is assumed to describe the fluctuation arising from the rhythms and the distribution of the rod cells along the retina, and *f*^*m, n*^(*t*) is the same global feedback function as in Equation (3). Upon $V_{i}^{m,n}$ reaching the threshold *V*_*th*_ from below, the *i*th neuron will emit an action potential at once and then the membrane potential is immediately reset to*V*_*r*_.

### Step 2. Decoding and Integration

The coming information from the rod cells is decoded into a binary image within the visual cortex. We explain it from two aspects. Firstly, the carrier of neural information transmission is spike impulse, so the encoded information should be in the form of a spike train instead of the continuous membrane potential. Secondly, note that rod cells play a minor role in color vision, which actually leads to loss of color in dim light (Purves, 2011; Owsley et al., 2016), so it is reasonable to assume that all the receiving spike trains can be transformed into a binary image. Let matrix (*Index*_*i*_)_*M* × *N*_ store the spiking information of the *i*th neuron at the encoding stage. Then, the corresponding binary image matrix (*Pic*_*i*_)_*M* × *N*_ decoded by the *i*th neuron can be written as

(10) $$\begin{array}{l} Pic_{i}\left(m,n\right)=\{\begin{array}{cc} \;0, & Index_{i}\left(m,n\right)=0;\\ \;255, & Index_{i}\left(m,n\right)=1. \end{array} \end{array}$$

With the decoded information from each neuron available, the visual cortex, as command center, will integrate all the information to form an overall gray image, which should be the picture we finally see in the dark surrounding. The idea of integration is inspired by boosting (Friedman, 2002). If each binary image is regarded as the output of the weak learner, the combination of the weak learners will be a strong learner and produce the gray image. We assume that the integration is in the way of linear superposition, namely,

(11) $$\begin{array}{l} Pic\left(m,n\right)=\frac{1}{N}\overset{k}{\underset{i=1}{\sum }}Pic_{i}\left(m,n\right) \end{array}$$

where (*Pic*)_*M* × *N*_ represents the integrated image.

We wish to put more emphasis on the validity of using the principle of stochastic resonance in our perception algorithm. It is well-known that noise is prevalent at the cellular level, and the level of the fluctuation in a neural system can be self-adjusted (Faisal and Selen, 2008; Durrant et al., 2011). What is more, distinct biophysical experiments (Douglass et al., 1993; Collins et al., 1996; Cordo et al., 1996; Levin and Miller, 1996; Pei et al., 1996; Borel and Ribot-Ciscar, 2016; Itzcovich et al., 2017; van der Groen et al., 2018) have shown that the benefit of noise can be utilized by biology. Thus, we assume that the human brain can select the perceived image of maximal contrast by means of the principle of stochastic resonance. The perceptive function of the brain is realized by neuron population, while the effect of stochastic resonance can be enhanced by uncoupled array or coupled ensemble; thus, our visual perception algorithm should be of some biological rationality.

**Figure 7:**
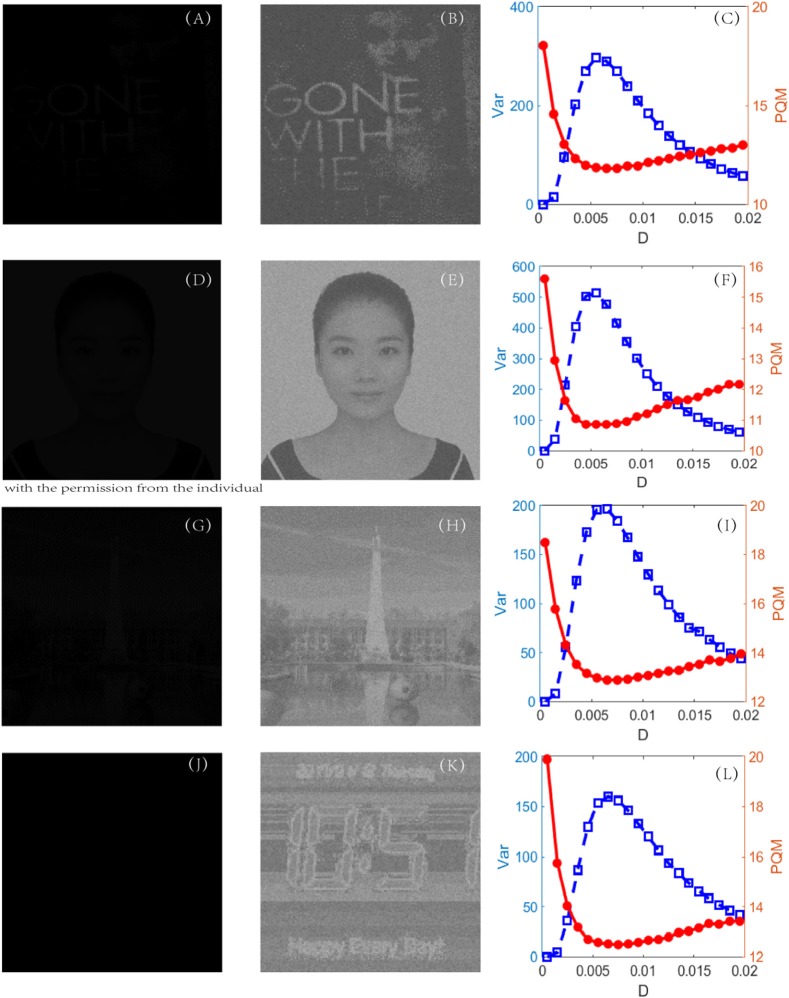
First column (**A**, **D**, **G** and **J**): original dark-input images; second column (**B**, **E**, **H** and **K**): enhanced images with best quality; and third column (**C**, **F**, **I** and **L**): dependence of variance (blue, square) and PQM (red, dot) on noise intensity, for each experiment. The parameters are set as *k* = 1,000, *V*_*T*_ = 0.1, *V*_*R*_ = 0, *G* = 0.12, τ_*s*_ = 0.05, τ_*D*_ = 0.01, and τ = 1. For each experiment, the location of the peak of variance is always near the location of the bottom of the PQM, indicating that variance helps in recognizing the best-quality image.

The procedure of the new algorithm is carried out in one unit of time by Euler integration with a step length of 0.01 time unit for all the detection experiments. The dark-input images were photos directly taken in a dark environment, such as that in Figure 7A, or artificially designed by compressing the original bright images into dark inputs, as shown in Figures 7D, G, J. The recognized images of the best quality, namely, the best enhanced images, are shown in the second column. During the experiments, it was found that some subtle key details, such as the quantifying index, the firing threshold, and the global feedback strength, need to be further explained.

#### Quantifying Index

**Figure 8:**
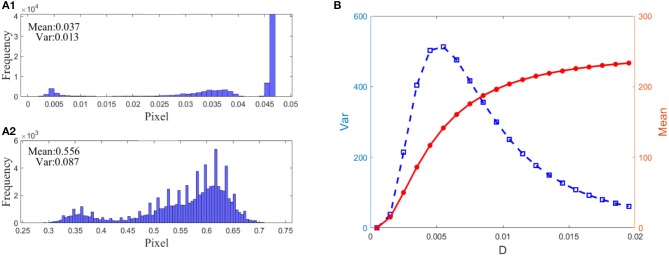
Demonstration of the advantage of variance over mean: **(A)** 1: normalized histogram of the dark input image in Figure 7D, **(A)** 2: normalized histogram of the best enhanced image in Figure 7E, **(B)** Dependence of the variance (blue square) and the mean (red dot) on noise intensity. As seen from **(A)** 1 and 2, the dark-input image and the best enhanced image differ in the histogram. Nevertheless, it is not applicable for picking up the image of the best contrast since the mean grows monotonically **(B)** as noise intensity increases, even l when the contrast of the detected image deteriorates again. By contrast, the bell-shaped change of variance is suitable.

To evaluate the quality of an image, in the image processing literature, the most frequently used indexes are the peak signal-to-noise ratio and the mean-square error, where some known reference images are required. The perceptual quality metric (PQM) (Wang et al., 2002), another quantifying index used in visual perception, can skillfully evade the reference images. The more that PQM is close to 10, the better the quality of the image is (Susstrunk and Winkler, 2003), but it tends to become flat near the optimal value, as shown in Figure 7. Since the flatness is not favorable for picking out the optimal noise intensity to get the best enhanced image, the objective here is to find a better quantifying index to assess the perceptual quality. The new index is found to be the variance of image. For a given image *U*_*M* × *N*_, the variance is defined by

$$\begin{array}{l} Var\left(U\right)=\frac{1}{{\left(M\times N\right)}^{2}}\overset{M}{\underset{i=1}{\sum }}\overset{N}{\underset{j=1}{\sum }}{\left(U\left(m,n\right)-\bar{U}\right)}^{2}, \end{array}$$

where Ū is the mean of the pixel matrix *U*_*M* × *N*_. The reason lies in the fact that this variance can reflect the heterogeneity among all the pixels. Intuitively, for a low-contrast image, the value of the variance will be quite low, but for a high-contrast image, the variance should take a much higher value. Figure 7 indeed verifies this reasoning. First of all, when the PQM is closer to 10, the variance curve will be nearer its peak. That is, the variance has the same capacity to identify which picture is the best in this task. Secondly, there is a sharp peak in the variance vs. the noise intensity curve so that one can easily detect an image with the best quality, namely, the best enhanced image. This is an advantage of the variance measure for the perceptual quality over the PQM measure, as shown in the third column of Figure 7. In addition, we note that the mean of the image is not suitable to be used as quantifying index. In fact, the mean of the image measures the luminance of an image, and it takes different values from the dark input and the best enhanced image to the blurred image due to excessive noise, but its value monotonically grows as noise intensity increases, as shown in Figure 8; thus, the mean is incapable of identifying the image with the best contrast as well. Undoubtedly, the comparison further emphasizes the applicability of the variance in visual perception.

#### Firing Threshold

**Figure 9:**
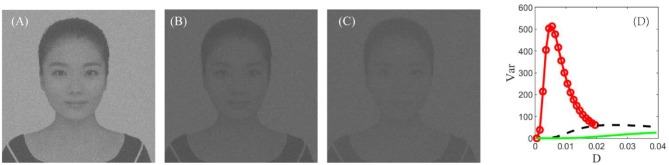
The best enhanced image under *V*_*T*_ = 0.1 **(A)**, *V*_*T*_ = 0.2 **(B)**, and *V*_*T*_ = 0.3 **(C)**, respectively. **(D)** Dependence of variance on the noise intensity under *V*_*T*_ = 0.1 (red, circle), *V*_*T*_ = 0.2 (black, dash), and *V*_*T*_ = 0.3 (green, solid). Other parameters are the same with Figure 7. This figure demonstrates that the best enhanced image is dependent on the spiking threshold, and thus choosing a suitable threshold is vital for image detection.

In real cortical activities, neurons can adopt a self-adaptive threshold strategy dependent on varying environments (Destexhe, 1998; Taillefumier and Magnasco, 2013) since the threshold has a direct impact on the neural electronic activity. We find that the threshold also has a large impact on the performance of the visual perception algorithm in Figure 9. The picture clearly shows that the choice of a suitable firing threshold is vital for the quality of the perceived image. Here the threshold is chosen according to the following rule. Firstly, find the frequency histogram of the dark image and denote the maximum pixel of the normalized histogram as max( *U*). Then, define the threshold by$V_{th}=10^{-1}ceil\;\left(10\text{max(}\;U\text{)}\right)$, where *ceil* (·) is the rounding function toward positive infinity. For example, the maximum pixel of the image in Figure 7D is max( *U*) = 0.05, as seen from Figure 8A1; accordingly, the threshold is taken as 0.1. It is worthy to remark that this kind of choice can guarantee that the distance between the base current and the firing threshold is minimized as far as possible, as suggested by the discussion following Figure 5.

#### Feedback Strength

**Figure 10:**
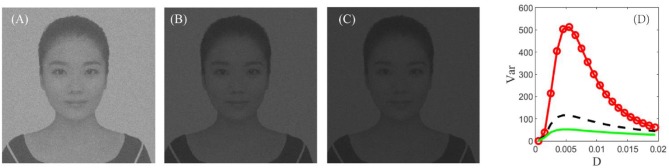
The best enhanced image under *G* = 0.12 **(A)**, *G* = 0 **(B)**, and *G* = −0.12 **(C)**, respectively. **(D)** Dependence of variance on the noise intensity under *G* = 0.12 (red, circle), *G* = 0 (black, dash), and *G* = −0.12 (green, solid). Other parameters are the same with Figure 7. Clearly, an excitatory feedback is the best among all the types of global feedback, and this implies that different rods should cooperate with each other when facing the same task.

In section stochastic resonance based image perception, it was demonstrated that, when the global feedback changes from the inhibitory type into the excitatory type, the peak of the signal-to-noise ratio can be improved as shown in Figure 4. This theoretical observation encourages us to check the influence of the feedback strength of the encoding stage on the enhanced images as illustrated in Figure 10. Evidently, the excitatory feedback leads to the best enhancement among all the cases, and thus one can fix the feedback strength to be positive as shown in Figure 7. We emphasize that this finding does not deny that inhibition plays an important role in visual perception (Roska et al., 2006). As we know, both excitation and inhibition exist in the retina (Rizzolatti et al., 1974). We assume that excitation is reflected by step 1 of our algorithm. That is, different neurons in the retina help each other in detecting the same target and exhibit the cooperative effect in a general homogenous network at the encoding stage. This cooperative effect helps the individuals of the network spike regularly, and certainly this effect is consistent with the description in Brunel (2000) which states that the neurons exhibit a regular state when excitation dominates inhibition.

## Conclusion

We have proposed a visual perception algorithm by combining the stochastic resonance principle of a global feedback network of integrate-and-fire neurons with the biophysical process for visual formation. The results can be summarized from the two closely related aspects. From the aspect of model investigation, we applied the technique of linear approximation and direct simulation to disclose the phenomenon of stochastic resonance in a global feedback network of integrate-and-fire neurons. It is demonstrated that both the spectral amplification factor and the output signal-to-noise ratio obtained from linear approximation are accurate when the size of the network is sufficiently large. Then, using the results derived from linear approximation, we found that positive feedback strength is beneficial for boosting the output signal-to-noise ratio, while a decreasing distance between the base current and the firing threshold can enhance the resonance effect. The theoretical observations are new, and they are also helpful for us to understand the working mechanism in rod neurons.

From the aspect of algorithm design, by applying the global feedback network (5) of integrate-and-fire neurons to simulate the perceptive process for rod cells, we have developed a novel visual perception algorithm. In the algorithm, the firing threshold is so critical that an inappropriate choice will lead to inefficiency in image enhancement. Under the inspiration of the theoretical finding that a decreasing distance between the base current and the firing threshold is favorable for stochastic resonance, we have proposed an explicit expression of a suitable firing threshold by referring to the histogram of the dark images. Moreover, we creatively introduced the variance of image rather than the perceptual quality metric as a more effective measure to examine the quality of the enhanced images. Massively numerical tests have shown that the biologically inspired algorithm is effective and powerful. We emphasize that the visual perception algorithm is a dynamical system based algorithm. We hope that it can be applied to relevant fields such as medical diagnosis, flight security, and cosmic exploration, where dark images are common. The algorithm also offers a good example of how the dynamical system research guides the neural engineering application. Following the success of this research, we will start to explore more interesting and important problems, such as the recovery of incomplete images, in the near future.

## Data Availability Statement

The raw data supporting the conclusions of this article will be made available by the authors, without undue reservation, to any qualified researcher.

## Author Contributions

YK guided and sponsored the research. YF did the simulation and algorithm implementation. YF worked out the initial draft, YK rewrote it, and GC made contribution in language polishing and general guide. The contributions from all the authors are important.

## Conflict of Interest

The authors declare that the research was conducted in the absence of any commercial or financial relationships that could be construed as a potential conflict of interest.
