# Supplementary material for: Stochastic Resonance Based Visual Perception Using Spiking Neural Networks
Source: Front Comput Neurosci. 2020 May 15;14:24. doi: 10.3389/fncom.2020.00024 (PMC7242793; doi:10.3389/fncom.2020.00024)
Supplement: Supplementary file 1 [file Data_Sheet_1.pdf]

Appendix: the derivation of spectral amplification factor and signal-to-noise ratio

According to the linear approximation theory, the output spike train of the Eq. (5) in the time domain has the form

$$y_i(t)=y_i^0(t)+R(\bar{\mu},D,t)*(f(t)-\langle f(t)\rangle_0+s(t)) \qquad \text{(A1)}$$

where $y_i^0(t)$ represents a realization of the spike train generated by the integrate-and-fire neuron obeying Eq. (A1) in the absence of the time-dependent perturbation, $*$ denotes the convolution, $\bar{\mu}=\mu+\langle f(t)\rangle_0$ is the base current, $R(\bar{\mu},D,t)$ is the base current and noise level-dependent linear response function, with Fourier transform called linear susceptibility to be explicitly given below, and $\langle f(t)\rangle_0=Gr(\bar{\mu},D)$ is the stationary average of the feedback term with the stationary firing rate $r(\bar{\mu},D)$, which can be found by solving the self-consistent relation [20,27,44]

$$r(\bar{\mu},D,V_T)=r_0(\bar{\mu}+Gr(\bar{\mu},D),D,V_T) \qquad \text{(A2)}$$

with $r_0(\mu,D,V_T)=(\tau_R+\sqrt{\pi}\int_{(\mu-v_T)/\sqrt{2D}}^{(\mu-v_R)/\sqrt{2D}} dz e^{z^2} erfc(z))^{-1}$. Introducing Fourier transform of the zero average spike trains as

$$\tilde{y}_i(\omega)=\frac{1}{\sqrt{T}}\int_0^T dt e^{i\omega t}(y_i(t)-r(\bar{\mu},D)).$$

Then, from Eq. (A1), one can get the linear response in the frequency domain as follows:

$$\tilde{y}_i(\omega)=\tilde{y}_i^0(\omega)+A(\omega,\bar{\mu},D,V_T)[\tilde{s}(\omega)+\frac{G}{N}F(\omega)\sum_{j=1}^{N}\tilde{y}_j(\omega)] \qquad \text{(A3)}$$

where $\tilde{y}_i^0(\omega)=\mathscr{F}[y_i^0(t)]$ represents a realization of the spiking output of the ith neuron in the frequency domain, $F(\omega)=e^{i\omega\tau_D}/(1-i\omega\tau_S)^2$ is the Fourier transform of the kernel in Eq. (2), and correspondingly $\frac{1}{N}F(\omega)\sum_{j=1}^{N}\tilde{y}_j(\omega)$ stands for the Fourier transform of the zero static mean synaptic feedback $f(t)-\langle f(t)\rangle_0$, and $A(\omega,\mu,D)=\mathscr{F}[R(\bar{\mu},D,t)]$ is the linear susceptibility given by [27]

$$A(\omega,\mu,D,V_T)=\frac{r(\mu,D)i\omega}{\sqrt{D}(i\omega-1)}\frac{\tilde{D}_{i\omega-1}(\frac{\mu-v_T}{\sqrt{D}})-e^{\gamma}\tilde{D}_{i\omega-1}(\frac{\mu-v_R}{\sqrt{D}})}{\tilde{D}_{i\omega}(\frac{\mu-v_T}{\sqrt{D}})-e^{\gamma}e^{i\omega\tau_R}\tilde{D}_{i\omega}(\frac{\mu-v_R}{\sqrt{D}})} \qquad \text{(A4)}$$

with $\gamma=[v_R^2-v_T^2+2\mu(v_T-v_R)]/4D$. Let

$$Y(\omega)=[\tilde{y}_1(\omega),\tilde{y}_2(\omega),\ldots,\tilde{y}_N(\omega)]^T,\quad Y_0(\omega)=[\tilde{y}_1^0(\omega),\tilde{y}_2^0(\omega),\ldots,\tilde{y}_N^0(\omega)]^T.$$

Rewrite Eq. (A3) as

$$Y(\omega)=Y_0(\omega)+A(\omega,\bar{\mu},D,V_T)\tilde{s}(\omega)\alpha+\frac{1}{N}GA(\omega,\bar{\mu},D,V_T)F(\omega)\alpha\alpha^T Y(\omega) \qquad \text{(A5)}$$

Then, by $\left\langle Y_0(\omega)\tilde{s}^*(\omega)\alpha\right\rangle=0$,

$$\left\langle Y(\omega)Y^H(\omega)\right\rangle=\left(I+\frac{1}{N}\frac{GA(\omega,\bar{\mu},D,V_T)F(\omega)\alpha\alpha^T}{1-GA(\omega,\bar{\mu},D,V_T)F(\omega)}\right)\left(\left\langle Y_0(\omega)Y_0^H(\omega)\right\rangle+\left|A(\omega,\bar{\mu},D,V_T)\right|^2\left\langle \tilde{s}(\omega)\tilde{s}^*(\omega)\right\rangle\alpha\alpha^T\right)$$
$$\times\left(I+\frac{1}{N}\frac{GA^*(\omega,\bar{\mu},D,V_T)F^*(\omega)\alpha\alpha^T}{1-GA^*(\omega,\bar{\mu},D,V_T)F^*(\omega)}\right)$$

where the superscripts H and $*$ denote transpose conjugate and conjugate, respectively, and $\alpha=[1,1,\ldots,1]^T$ is a constant auxiliary vector.

Note that for a homogeneous network, the population activity $y(t)=\frac{1}{N}\sum y_i(t)$ is of central importance. From the definition of spectral density $G_{yy}(\omega)=\frac{1}{N^2}\alpha^T\left\langle Y(\omega)Y^H(\omega)\right\rangle\alpha$, one can obtain

$$G_{yy}(\omega)=\left(1+\frac{GA(\omega,\bar{\mu},D,V_T)F(\omega)}{1-GA(\omega,\bar{\mu},D,V_T)F(\omega)}\right)\left(\frac{1}{N^2}\sum_{i=1}^{N}\sum_{j=1}^{N}\left\langle y_i^0(\omega)y_i^{0*}(\omega)\right\rangle+\left|A(\omega,\bar{\mu},D,V_T)\right|^2\left\langle\tilde{s}(\omega)\tilde{s}^*(\omega)\right\rangle\right)\left(1+\frac{GA^*(\omega,\bar{\mu},D,V_T)F^*(\omega)}{1-GA^*(\omega,\bar{\mu},D,V_T)F^*(\omega)}\right)$$
$$=\frac{\left|A(\omega,\bar{\mu},D,V_T)\right|^2\left\langle\tilde{s}(\omega)\tilde{s}^*(\omega)\right\rangle+\frac{1}{N}S_0((\omega,\bar{\mu},D,V_T))}{\left|1-GA(\omega,\bar{\mu},D,V_T)F(\omega)\right|^2}. \qquad \text{(A6)}$$

Let $S_1(\omega)=\frac{|A|^2}{|1-GAF|^2}\left\langle\tilde{s}\tilde{s}^*\right\rangle$ be the spectral density of the signal component, and $S_2(\omega)=\frac{1}{N|1-GAF|^2}S_0(\omega,\bar{\mu},D,V_T)$ with

$$S_0(\omega,\mu,D,V_T)\overset{\Delta}{=}\left\langle\tilde{y}_i^0(\omega)\tilde{y}_i^{0*}(\omega)\right\rangle=r(\mu,D)\frac{|\tilde{D}_{i\omega}(\frac{\mu-v_T}{\sqrt{D}})|^2-e^{2\gamma}|\tilde{D}_{i\omega}(\frac{\mu-v_R}{\sqrt{D}})|^2}{|\tilde{D}_{i\omega}(\frac{\mu-v_T}{\sqrt{D}})-e^{\gamma}e^{i\omega t_R}\tilde{D}_{i\omega}(\frac{\mu-v_R}{\sqrt{D}})|^2} \qquad \text{(A7)}$$

representing spectral density of fluctuations, then $G_{yy}(\omega)=S_1(\omega)+S_2(\omega)$. With the spectral density $G_{yy}(\omega)$ available, the spectral amplification factor and signal-to-noise ratio can be obtained accordingly, as shown in Eqs.(6) and (7).
